# Infant sensory gating and a developmental cascade to autistic traits and anxiety

**DOI:** 10.1038/s41386-025-02253-6

**Published:** 2025-10-15

**Authors:** Rebecca F. Schwarzlose

**Affiliations:** Department of Psychiatry, Washington University in St. Louis School of Medicine, St. Louis, MO USA

**Keywords:** Human behaviour, Perception, Predictive markers

## Abstract

Disruptions to the infant sensory environment can have lasting effects on neural response properties and behavior in both humans and animals. Recent work has begun to highlight an additional factor in infant sensory experience: differences in inhibitory signaling and sensory gating. Converging work from human and animal studies has begun to implicate a developmental cascade by which impaired sensory gating during a sensitive period of neonatal neurodevelopment promotes a phenotype of sensory over-responsivity, autistic traits, anxiety, and other psychiatric challenges. In this Review, I propose a model for this developmental cascade and highlight how differences in infant sensory responsivity represent an important intermediate phenotype for research, screening, and supportive intervention.

## Introduction

At any given moment, the human brain is inundated with signals from more than 210 million sensory receptors [1]. We have this wealth of sensory receptors because our survival depends on the ability to detect and respond to important cues in our environment. Unfortunately, the brain can only process, perceive, and attend to a fraction of these inputs at a time. Survival and success depend as much on ignoring irrelevant sensory inputs as they do on perceiving the relevant ones [2, 3].

Evolutionarily conserved neural mechanisms meet this challenge by filtering out uninformative sensory signals. These mechanisms, including *habituation* (Box 1) and *prepulse inhibition*, are collectively referred to as sensory gating [3]. They rely on inhibitory signaling via γ-aminobutyric acid (*GABA*), the primary inhibitory neurotransmitter in the postnatal brain. Together, these mechanisms drive adaptive modulation of sensory processing to support an organism’s moment-to-moment survival needs. Although these mechanisms are ubiquitous across the animal kingdom, they are still subject to interindividual variation. In this Review, I describe a common behavioral phenotype, *sensory over-responsivity (SOR)*, that is related to impaired sensory gating and risk for psychiatric disorders. I highlight converging work from human and animal studies pointing to inhibitory dysfunction and impaired sensory gating in infancy as catalysts for a developmental cascade of SOR, autistic traits, and anxiety in later life.

Box 1 Relevant terms Sensory Over-Responsivity (SOR): SOR is a behavioral phenotype characterized by patterns of extreme negative reactions to typically innocuous stimuli.

γ-aminobutyric acid (GABA): GABA is the main inhibitory neurotransmitter of the mature central nervous system.

NKCC1 transporter: the Na-K-2Cl cotransporter isoform 1 (NKCC1) is the immature form of the chloride transporter that increases intracellular chloride ion concentrations, making the action of GABA depolarizing (excitatory).

KCC2 transporter: the K-Cl cotransporter isoform 2 (KCC2) is the mature form of the chloride transporter that decreases intracellular chloride concentrations, making GABA hyperpolarizing (inhibitory).

Prepulse inhibition (PPI): a form of sensory gating in which presentation of a weak stimulus (prepulse) attenuates the neural and behavioral response to a subsequent strong and unexpected stimulus. For example, presenting an auditory prepulse before a loud sound attenuates the startle response to the loud sound.

Habituation: a form of sensory gating in which both neural and behavioral responses to a repeating sensory stimulus diminish over successive stimulus presentations.

Excitation-to-inhibition (E/I) balance: effective neural processing requires a balance of excitatory neurotransmission (via glutamate) and inhibitory neurotransmission (via GABA). Adequate function of both excitatory and inhibitory circuits is required to support neural function and avoid unchecked excitation, including seizures.

Behavioral inhibition (BI): a form of anxious temperament characterized by greater vigilance to and avoidance of unfamiliar people, objects, and situations. BI can be assessed beginning around 12 months of age.

Negative reactivity: a form of anxious temperament that can be observed in infants based on their responses to novelty. Negative reactivity is characterized by high negative affect and low positive affect to novel people, places, and objects.

## Sensory over-responsivity

SOR is a behavioral phenotype with moderate to high heritability [4] characterized by strong, negative reactions to typically innocuous stimuli. It affects approximately 10–20% of school-age children in the population [5–7], with far higher rates (50–95%) among children with common psychiatric and neurodevelopmental disorders [5, 8, 9]. SOR was recently added to the diagnostic criteria for autism spectrum disorder [10, 11], however, it is associated with both autistic traits and symptoms of other common psychiatric conditions across diverse child samples [5, 6, 12–14]. It is also positively associated with premature birth and socioeconomic disadvantage [5, 6, 15, 16]. Reliable measures of SOR are difficult to obtain in infants younger than 12 months of age because of their limited behavioral repertoires; however, longitudinal studies support its relative stability across childhood [16–18]. Even at the 12-month timepoint, measures of SOR may provide information about clinical risk; scores at 12 months of age are higher in infants who go on to receive a diagnosis of autism as toddlers, compared with those who do not [19].

Although SOR is broadly defined as a pattern of severe and atypical negative reactions to otherwise harmless stimuli, there is variation across individuals in how SOR is expressed. A given individual may exhibit SOR for stimuli in a single sensory modality (e.g., only to certain sounds) or across different sensory modalities. Often, individuals show a more complex pattern of atypical sensory responding that includes SOR, sensory under-responsivity (SUR; failure to register and respond to salient stimuli), and sensory seeking (atypical engagement in sensory self-stimulation) in response to different stimuli. For example, many autistic children exhibit SOR for irrelevant background noises (e.g., sounds of vacuum cleaners or flushing toilets) but SUR for important cues like someone yelling their name [8, 20, 21]. At first glance, these complex patterns of sensory responding might appear contradictory. However, they are consistent with a broader observation: that autistic children tend to allocate attention to sensory stimuli in their environment in an atypical fashion. For instance, they tend to look less at social stimuli like eyes and more at nonsocial stimuli like objects or backgrounds than their typically developing peers [22–24]. These patterns may reflect the fact that sensory inputs compete for neural processing, attention, and awareness [25, 26]. Sensory gating adjudicates this perceptual competition, steering perception and subsequent processing toward behaviorally relevant stimuli. By affecting which sensory stimuli are processed during developmentally programmed refinement of sensory circuits, sensory gating during infancy may influence how children allocate attention and respond to sensory stimuli later in childhood. Additional empirical research is needed to clarify how early sensory gating might impact later attention allocation toward sensory stimuli.

In addition to its well-established link with autism, SOR is overrepresented among children with many common neuropsychiatric conditions of childhood, including anxiety disorders, depression, and attention-deficit/hyperactivity disorder (ADHD) [5–7, 27]. However, high rates of co-morbidity and symptom co-occurrence in pediatric psychiatry make bivariate SOR-symptom associations hard to interpret. Using multivariate analyses to account for symptom co-occurrence across six pediatric conditions (anxiety disorders, depression, ADHD, oppositional defiant disorder, and conduct disorder) in five separate datasets, we have shown that SOR is reliably associated with both autistic traits and anxiety symptoms, but not with symptoms of the other modeled conditions [28, 29]. These effects are consistent across sample characteristics, including age and community versus clinical recruitment. Although these correlational results cannot establish causality, they implicate SOR as a possible latent transdiagnostic marker of risk for two neuropsychiatric conditions of childhood.

Findings from longitudinal studies generally support the idea that SOR precedes and forecasts emerging anxiety. In one community sample, preschool SOR (ages 2 to 5 years) was associated with increases in anxiety symptoms at 6 years, controlling for preschool anxiety [6]. This effect was unidirectional, as preschool anxiety was not associated with increased SOR at age 6, when controlling for preschool SOR. Similarly, in a large community sample of preadolescents, both mild and severe SOR were associated with increased anxiety symptoms 1 year later, controlling for concurrent psychiatric symptoms and autistic traits [5]. In a sample of toddlers enriched for familial likelihood of developing autism or ADHD, SOR at 14 months was associated with increased anxious temperament at 24 months [14]. Again, this relationship was unidirectional; anxious temperament at 14 months was not associated with increased SOR at 24 months. The same pattern was found among toddlers with autism or pervasive developmental disorder diagnoses across two timepoints spaced 1 year apart; initial SOR was associated with increased later anxious temperament, whereas initial anxious temperament was not associated with increased later SOR [30]. These studies are observational and cannot establish causality. Still, they collectively support the conclusion that SOR precedes and predicts worsening anxiety symptoms, both in neurotypical and autistic children.

It may be helpful to consider this evidence linking SOR and anxiety in the context of prevailing hypotheses about anxious temperament and anxiety risk. In particular, decades of developmental research on early-life anxious temperament have identified atypical, negative responses to novel people, places, and objects in infancy (i.e., *negative reactivity*) and early childhood (i.e., *behavioral inhibition*) as potent risk factors for later anxiety disorders [31–38]. This line of work has shown that early anxious temperament and later pediatric anxiety are characterized by hypervigilance and biased allocation of attention to potential threats [39–42]. Several studies (described below) suggest that anxious temperament is often associated with impaired sensory gating.

How do we reconcile the existing work on anxious temperament with the present findings linking SOR to anxiety? One possibility is that SOR and anxious temperament act as independent risk factors for emerging anxiety. Alternately, some conceptual frameworks have posited a direct relationship between them [43, 44]. For example, it has been proposed that the hypervigilance associated with anxious temperament and anxiety disorders may predispose children to react negatively to certain sensory stimuli (i.e., exhibit SOR) [43]. Another framework posits that diminished sensory gating—specifically, habituation—to novel stimuli in early infancy may cause children to react negatively to novel objects and situations later in childhood (i.e., exhibit anxious temperament) [44]. There are clear similarities between the two behavioral constructs in that they are both characterized by atypical negative or avoidant reactions to certain environmental triggers. However, there are also important distinctions. Whereas responses in anxious temperament are linked to stimulus novelty (a property that changes with repeated exposure), responses in SOR are linked to stimulus-defined features (stimulus type or modality). The relationship between these phenotypes is an important open question that can only be answered with empirical evidence from additional research.

## Sensory gating in infancy

The term sensory gating refers to both the neural mechanisms of filtering out uninformative stimuli and its physiological effects (e.g., lower heart rate or skin conductance in response to sensory stimulation). Scientists can now obtain neural measures of sensory gating in humans with several powerful, non-invasive neuroimaging techniques, including electroencephalography (EEG) and functional MRI, which provide good temporal and spatial resolution, respectively.

Research using neuroimaging and physiological techniques has consistently found reduced neural habituation to auditory, tactile, and visual stimuli in children and adults with autism [45–54]. Results from two studies that separately analyzed this effect in relation to children’s SOR symptoms suggest the effect is related to SOR symptoms, rather than autism diagnosis or autistic traits per se; however, more studies are needed to conclusively answer this question [49, 51]. Sensory gating deficits, and specifically impaired habituation, have also been observed in strains of transgenic mice, fruit flies, and nematodes containing genetic mutations associated with autism in humans [55–59].

A central challenge for studying how sensory gating relates to autistic traits and anxiety risk is one of timing. In the first few months of life, infants lack the behavioral repertoire to support reliable behavioral assessment of autistic traits and anxious temperament. Diagnoses for autism can be given as early as the second year of life, whereas pediatric anxiety disorder diagnoses are typically made during or after preschool years [60, 61]. Therefore, labor- and time-intensive longitudinal studies are required to establish relationships between early sensory gating and later autism and anxiety.

A few longitudinal studies to date have examined this question using neural or physiological measures of infant sensory gating. Most of these studies assess sensory gating in infancy with EEG while a sleeping infant is played repeating stimuli (typically, presented in pairs); sensory gating is measured by the reduction of the neural response to the second of the paired stimuli. Measures of sensory gating derived from this paradigm appear to be reliable in early infancy [62] and demonstrated stability from infancy to later childhood in a small sample [63], although more work with larger samples is needed. In a sample of children at high familial likelihood for developing autism, reduced sensory gating to repeated sounds was found in 8-month-old infants who went on to receive an autism diagnosis, relative to those identified as typically developing toddlers, at 3 years of age [64]. In another sample enriched for familial likelihood of autism or ADHD, reduced gating of repeated tactile stimulation in 10-month-old infants was positively associated with children’s autistic traits at 2 years of age [65]. A more extensive body of work capitalizing on a hearing test administered universally to newborns, the auditory brainstem response (ABR) screening, finds longer ABR latencies in neonates who go on to develop autism [66–70]. Although the ABR results do not speak to sensory gating per se, they show that measurable neonatal differences in neural sensory processing are related to autism likelihood.

Few longitudinal studies have focused on sensory gating in relation to risk for anxiety or emerging anxiety symptoms; however, they provide preliminary support for a relationship. In one of these, reduced sensory gating to repeated auditory stimulation was found in infants born to mothers with a history of anxiety disorders, relative to mothers with no history of anxiety [71]. In a subset of the same cohort, children categorized as exhibiting reduced sensory gating in infancy (relative to those who exhibited robust sensory gating) scored higher on anxiety and ADHD-related problems, based on parent report at 3 years of age [72]. Another study found that neonatal physiological habituation to repeated auditory stimulation at 2 weeks of age (but not 6 weeks), as measured by changes in heart rate, was negatively associated with observational measures of anxious temperament obtained from the children at 14 months of age [44].

It is important to note that the infant studies described here vary in their procedures and outcome measures. Most have modest sample sizes, requiring confirmation through replication in larger samples. Yet taken together, these findings provide preliminary evidence that diminished infant sensory gating may be associated with emerging autistic traits and anxious temperament later in childhood. These studies of human infants have been limited to non-invasive observational measures and correlational analyses for obvious ethical reasons. In the next section, I review evidence from model systems that provides deeper insights into the causal, neural mechanisms supporting early sensory gating and its lasting impact on behavior.

## Inhibition in infant sensory gating

The perinatal period is a time of dramatic change in the function and properties of developing sensory circuits. It coincides with the onset of GABA-mediated inhibition, an evolutionarily conserved process that is central to neurodevelopment [73]. Prenatally, widespread expression of the chloride ion transporter *NKCC1* causes GABA to function as an excitatory rather than inhibitory neurotransmitter [74]. Prenatal sensory circuits primarily amplify spontaneous, intrinsically generated signals from sensory receptors; these signals refine sensory circuits and sculpt topographic brain maps [75]. Sensory and hormonal triggers around the time of birth ramp up the expression of the mature chloride ion transporter KCC2, which switches the action of GABA from excitatory to inhibitory [76]. This switch generates feedforward inhibition, shifting sensory circuits to their mature role of conveying external sensory signals rather than intrinsic ones. At the same time, infants enter a period of dramatic refinement of sensory circuits based on inputs from their postnatal sensory environment [2, 77, 78]. Alterations in the features of this sensory environment can produce lasting changes in sensory brain maps and the underlying response properties of constituent neurons. For example, monocular visual deprivation in the postnatal period reduces cortical representation of inputs from the deprived eye [79] and has lasting impacts on visual acuity even after binocular inputs are restored (as in late cataract removal) [80].

The perinatal emergence of inhibitory circuits is instrumental to sensory gating and regulates the flow of information about external sensory stimuli to the developing central nervous system (CNS). Perinatal disruptions such as prenatal maternal immune activation and perinatal stress can affect the timing of this process in rodent models [81, 82]. Premature birth is also likely to affect this timing, although conclusive evidence documenting this effect in humans is hampered by the challenges of measuring this switch in vivo. Moreover, directions of these effects may depend on details such as gestational age at birth, cause of premature birth, and atypical postnatal sensory environments such as neonatal intensive care units.

Inhibitory function and related indices of neural *excitation-to-inhibition (E/I)* ratios have featured prominently in existing models of autism and SOR [83, 84]. Clues from magnetic resonance spectroscopy [85–87] and from high rates of epilepsy in children with autism [88] are broadly consistent with the hypothesis that autism is related to E/I imbalance and, specifically, reduced inhibitory function. Additional evidence has come from research with several transgenic animal models of neurodevelopmental disorders related to autism. Although the neural loci and cellular bases of abnormalities differ by genetic mutation, these animals tend to exhibit reduced neonatal GABAergic function in sensory circuits, resulting in impaired neonatal and juvenile sensory gating, social impairments, and anxiety-like behavior such as avoidance of novel or exposed spaces [57–59, 89–92]. Scientists assess anxiety-like behavior in mice with behavioral assays like the open-field test, in which the animal is placed in a novel chamber. Reduced exploration of the novel chamber and, particularly, the exposed center of the chamber, are coded as anxiety-like behaviors [59]. As such, they resemble behavioral inhibition assessments for infants, which identify BI based on a child’s reduced exploration of novel environments and unwillingness to approach novel objects or people [33, 93].

Separate lines of research with transgenic mice illustrate how impaired neonatal inhibitory function in sensory circuits can trigger a lasting neurodevelopment cascade. A striking example comes from research with transgenic mice harboring a mutation in Mecp2 that causes the neurodevelopmental disorder Rett syndrome in humans [59, 89]. Rett syndrome tends to co-occur with autism and atypical sensory reactivity, including SOR; the corresponding genetic mutation in mice also produces sensory gating impairments (including reduced habituation), social impairments, and anxiety-like behaviors. A series of elegant studies showed that these mice have reduced postnatal GABA-mediated presynaptic inhibition, or insufficient gating, of tactile inputs from peripheral somatosensory neurons to the spinal cord and brain [59]. In addition to the animals’ behavioral deficits by adulthood, they have fewer parvalbumin-containing (PV+) inhibitory interneurons in the primary somatosensory cortex (S1), resulting in an excessive E/I ratio [89]. Remarkably, limiting expression of the Mecp2 mutation to peripheral somatosensory neurons (i.e., not in the CNS) is sufficient to trigger the behavioral impairments and E/I imbalance in primary somatosensory cortex, but only when this expression occurs in the postnatal period; expression in adulthood produces sensory gating impairments without social impairments or anxiety-like behaviors [59]. Conversely, postnatal administration of a GABA agonist that boosts inhibitory function exclusively in the peripheral nervous system rescues the animal from anxiety-like behaviors, E/I imbalance in the primary somatosensory cortex, and some social impairments in adulthood [89]. Similar effects were found in transgenic mice harboring the mutations in Gabrb3 and Shank3 that respectively cause Angelman’s syndrome and Phelan-McDermid syndrome—two other neurodevelopmental disorders associated with autism—in humans [59, 89]. Collectively, these studies demonstrate that deficient inhibitory gating of sensory signaling specifically during a postnatal critical period of development can produce long-term cortical E/I imbalance, social impairments, and anxiety-like behavior characterized by novelty avoidance [94].

Another illuminating set of studies in transgenic mice illustrates how E/I imbalance in the postnatal period can lead to irreversible alterations in cortical microcircuitry. In transgenic mice with mutations in the Fmr1 gene that causes Fragile X syndrome, decreased excitability of neonatal PV+ inhibitory interneurons in S1 triggers a dramatic, permanent programmed cell death of these interneurons in the first two postnatal weeks [58]. These animals also exhibit impaired sensory gating, including reduced sensory adaptation and PPI [57], and altered S1 cortical maps (specifically, enlarged S1 whisker representation) from the third postnatal week to adulthood [95]. Mice with selective disruption of Fmr1 only in PV+ inhibitory interneurons show the same pattern of social deficits and anxiety-like behavior as those with germline mutations that affect all cells, whereas selective disruption of the gene in a different class of inhibitory interneurons did not produce this behavioral pattern [96]. Finally, chronic postnatal administration of bumetanide, an NKCC1 antagonist that promotes the postnatal GABAergic inhibitory switch, reduces tactile hypersensitivity, rescues S1 neural adaptation, and restores a typical S1 cortical map [95, 97]. Taken together, these studies illustrate a different route by which immature or insufficient neonatal inhibitory function can trigger lasting changes in E/I balance, sensory cortical representation, and, ultimately, social and anxiety-like behavior.

It is important to note that the animal models described above are not representative of most autistic children, not to mention children with anxiety disorders, from a genetic perspective. Single-gene variants of large effect make up a small minority (about 5%) of autism cases [98]. The genetic bases of most autism cases and ostensibly all cases of anxiety disorders are thought to be polygenic, or related to the cumulative small effects of many common gene variants [98, 99]. These transgenic mice, and the human conditions after which they are modeled, also differ from one another genetically, physiologically, and phenotypically. However, these animal models provide an unparalleled window into neurodevelopment at the cellular level and can establish neurodevelopmental causality in ways that could never be ethically undertaken with humans. Despite differences between models, they exhibit several striking similarities. Namely, they show that disruption of inhibitory function in peripheral or central sensory circuits during a critical period of postnatal development (but not in adulthood [100]) converges to produce a constellation of physiological and behavioral characteristics similar to those observed in humans, including impaired sensory gating, E/I imbalance in sensory cortex, anxiety-like behaviors, and social impairments. Perhaps most importantly, they demonstrate that postnatal inhibitory dysfunction causes these long-term outcomes, whereas neonatal genetic or pharmacologic intervention to restore inhibitory function prevents them. These findings establish causality and raise the possibility that targeted intervention during specific early windows of development could uniquely protect children from developing certain sensory, social, and anxiety challenges.

Notably, studies have documented a delay in the GABAergic switch among humans and transgenic mice with autism-related genetic mutations, as reviewed elsewhere [101]. Because assessing this switch entails invasive procedures, most of this evidence comes from animal models. However, a small study in humans did find lower levels of the mature chloride channel, KCC2, and lower ratios of mature to immature channels (KCC2/NKCC1) in the cerebrospinal fluid of children with Rett Syndrome, relative to controls without a Mecp2 mutation [102]. Moreover, administration of the NKCC1 inhibitor bumetanide restores typical E/I balance in transgenic mice [103]. Delays in the timing of the GABAergic switch may be functionally equivalent to deficits in GABAergic transmission in driving up the E/I ratio and disrupting sensory gating during this window of postnatal neurodevelopment. Collectively, these studies highlight the complexity of inhibitory maturation in the neonatal period and its relevance to the phenotypic constellation of attenuated sensory gating, anxiety-like behavior, and social impairments.

## Reduced inhibition in sensory circuits: trigger of a neurodevelopmental cascade?

The evidence reviewed above implicates reduced inhibitory function and impaired sensory gating in infancy as risk factors for SOR and emerging neurodevelopmental and psychiatric symptoms. But how might these early differences lead to the specific constellation of SOR, anxiety, and social impairments? Here, I propose a neurodevelopmental cascade based on the existing evidence from studies of humans and animal models. This reduced inhibition in sensory circuits (RISC) cascade begins with insufficient inhibition of feedforward sensory signaling and impaired sensory gating in infancy. The disruption coincides with a developmental period of sensory circuit refinement via programmed cell death, synaptic pruning, and assembly of perineuronal nets [104]. During this window, passive exposure to sensory stimuli in the environment, or lack thereof, serves as a sensory curriculum to the developing brain [105]. A large body of work on developing animals has shown that alterations to this curriculum, whether in tactile, auditory, or visual domains, can produce lasting changes in neural circuits that support perception and stress responses [77–79, 104, 106, 107]. By altering the impact of environmental stimuli on the developing CNS during this period, impaired sensory gating is positioned to alter this sensory curriculum in ensuing stages of neurodevelopment.

**Fig. 1:**
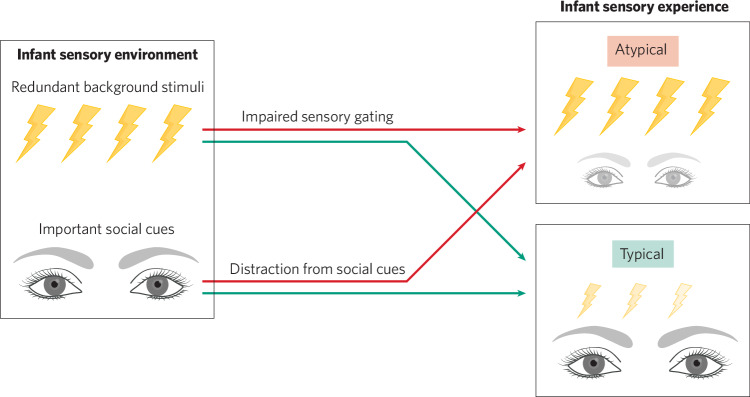
How sensory gating disrupts infant sensory inputs to the developing CNS. An infant’s sensory environment simultaneously contains both irrelevant stimuli, like background noises, and important social cues that promote social interaction and learning. Impaired sensory gating produces excessive processing of irrelevant stimuli, which may: (1) cause the infant to experience repeated, context-inappropriate sensory distress and (2) compete with infants’ processing of dynamic social cues, reducing infant looking at and attention to these important stimuli.

Figure 1 illustrates how two infants in the same sensory environment can receive radically different sensory inputs depending on their sensory gating abilities. Virtually every environment contains a multitude of innocuous sensory stimuli, from the din of ambient noises and glare of overhead lights to the pressure of clothing or surfaces against one’s skin. Infants with impaired sensory gating are unable to adaptively filter these stimuli out. The resulting sensations can both produce uncontrollable sensory discomfort (Fig. 1, top) and distract a child’s attention away from more important stimuli in the environment, like social cues (Fig. 1, bottom). In mature animals and human adults, uncontrollable aversive experiences have been shown to induce anxiety, depression, and helplessness [108]. Similarly, SOR may promote anxiety in a cumulative fashion across infancy and childhood [5, 6, 14, 30, 43] because it exposes children to repeated, uncontrollable sensory discomfort that others around them do not share or understand. A child experiencing repeated discomfort from stimuli that others barely notice might learn to be vigilant and wary of new environments. As such, impaired sensory gating and SOR may be one factor promoting anxious temperament and, eventually, anxiety.

In processing irrelevant stimuli, infants with impaired sensory gating and SOR face another challenge: what they fail to process and perceive. From looking behavior to attention, perception is a competitive process [25, 109, 110]. In this framework, excessive processing of irrelevant stimuli also tends to reduce processing of simultaneous relevant stimuli in the environment. Infant attention is typically drawn to dynamic stimuli like human faces that provide the building blocks for social learning and interaction [105, 111, 112]. Notably, infants who go on to meet diagnostic criteria for autism as toddlers tend to show divergent looking behavior and, specifically, reduced looking at eyes, relative to their typically developing peers [22, 23, 109]. If SOR reduces infant orienting to social stimuli and processing of social cues during formative periods of social development, this might explain why SOR is associated with autistic traits across the lifespan [8, 113–115].

**Fig. 2:**
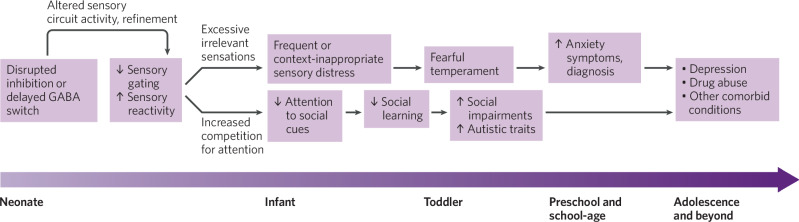
The reduced inhibition in sensory circuits (RISC) model. The model shows how disrupted inhibitory function in early infancy may lead to a cascade of impaired sensory gating, increased sensory reactivity, fearful temperament, social impairments, and emerging neurodevelopmental and psychiatric symptoms.

Considering the impact of impaired sensory gating on infant sensory experience provides a plausible pathway whereby disrupted inhibitory function and sensory gating can lead to the specific constellation of SOR, anxiety, and social impairments observed in human and animal studies. A preliminary model of this pathway is shown in Fig. 2. As the work from transgenic animal models shows, different genetic mutations that interfere with inhibitory function at different stages of feedforward sensory processing can converge in triggering the RISC cascade and producing the same constellation of behavioral phenotypes. The common theme among these triggers appears to be their net effect of diminished sensory gating in early infancy. It is also important to note that RISC is presumably not the only pathway to autism or anxiety disorders, both of which are complex, heterogeneous conditions that can occur independently of one another [116–118].

Many pivotal developmental processes exhibit canalization, which means they are buffered to environmental or genetic perturbations [119–121]. RISC would seem to represent an exception to canalization, in that it leads to an alternate developmental trajectory and outcome. This exception could reflect a period of vulnerability due to the complex and rapid changes in inhibitory circuit function and heightened neuroplasticity in the neonatal period. In addition, this alternate developmental outcome does not prevent infants from surviving to adulthood or from having offspring, and may therefore escape evolutionary pressure from natural selection. Nonetheless, individuals with SOR describe it as an ongoing source of distress and functional impairment [10]. Other outcomes associated with RISC, including anxiety disorders and common secondary conditions such as major depression, are detrimental to human health and well-being. Consequently, advances in identifying and mitigating risk for this cascade during a sensitive period of development would have the potential to alleviate suffering and reduce lifetime mental health burdens.

## Future research directions

Given evidence for a neonatal sensitive period for RISC in animal models, it will be important to empirically establish whether a similar sensitive period exists for humans. If so, this sensitive period may close before SOR can be behaviorally identified in infants and well before the emergence of anxiety and other psychiatric symptoms. Interventions may have the greatest effect if they are timed to coincide with the neonatal period when neural plasticity is greatest. These interventions might include treatment with pharmacologic agents that support inhibitory action or maturation. The diuretic bumetanide, mentioned above, promotes the GABAergic switch by inhibiting NKCC1 and has shown promise in reducing neural and behavioral outcomes of RISC in transgenic mouse models of autism [97, 103]. However, randomized controlled trials of bumetanide in older children (school-age and adolescent) have failed to demonstrate reductions in autism symptom severity [122–124]. Moreover, trials for its use as an add-on to the GABA agonist phenobarbital for treating neonatal seizures have proven ineffective and identified potential adverse side effects in high-risk infants [125, 126]. Considerable future research would be needed to determine whether candidate pharmacologic agents are safe and confer protective effects when administered during specific developmental epochs.

Candidate interventions might also include environmental enrichment or targeted changes to the sensory environment in early development. For example, housing neonatal mice in enriched environments (e.g., larger cages containing other mouse dams and pups, varied nesting materials, and novel objects) promotes the GABAergic switch to inhibition [127, 128] and rescues Mecp2 mutant mice from later anxiety-like behavior in open-field tests [129]. In the case of dogs, environmental enrichment through exposure to a variety of people and sensory stimuli during a critical period in the first 3 months of life is protective against tactile over-responsivity, anxiety, and inhibition around humans later in life [130, 131]. Analogous environmental enrichment for human infants might include exposure to a wider variety of people, places, and sensory stimuli in early infancy.

If supportive intervention is possible, another challenge will be identifying the infants who could benefit from it. Neonates have a limited behavioral repertoire and their primary method for communicating distress—crying—is nonspecific. Although infant crying and fussiness could signal sensory distress and are predictors for later internalizing symptoms and behavioral problems [132–134], infants also cry due to gastrointestinal discomfort or for myriad other reasons. Therefore, a major hurdle to early intervention would be developing reliable and scalable screening techniques to identify infants at risk. Similar hurdles have been overcome in detecting sensory organ dysfunction in the neonatal period, resulting in universal newborn screening for hearing and vision that permits intervention or accommodation during sensitive periods of neurodevelopment [135, 136]. Additional research is needed to investigate the RISC cascade in humans and develop innovative solutions for identifying and mitigating risk during this period of heightened neuroplasticity.
